# Fournier's gangrene after male circumcision: A case report

**DOI:** 10.1016/j.eucr.2025.102950

**Published:** 2025-01-15

**Authors:** Isobel A. Bowring, Lotte Bouwman, Kim van Putten, Joris J. Blok

**Affiliations:** a Department of Surgery, Groene Hart Ziekenhuis, Gouda, the Netherlands; b Department of Urology, Groene Hart Ziekenhuis, Gouda, the Netherlands; c Department of Surgery, Leiden University Medical Center, Leiden, the Netherlands

**Keywords:** Case report, Fournier's gangrene, Necrotizing soft tissue infection, Circumcision

## Abstract

Necrotizing soft tissue infection (NSTI) is a rare, life-threatening emergency. Fournier's gangrene (FG), a type of NSTI affecting the perineal, genital, or perianal region, requires prompt and aggressive treatment and has a multifactorial aetiology. However, cases of FG in patients without predisposing factors, as a complication of an elective procedure, are seldom reported. In this report, a 32-year-old patient without prior medical history presented with a NSTI after elective circumcision. Patient was successfully treated with multiple surgical debridement's, antibiotic therapy and hemodynamic support. Healthcare providers must remain vigilant, as FG has significant morbidity and mortality and early intervention is crucial.

## 1 Introduction

Fournier's gangrene (FG), first described by Jean Alfred Fournier in 1883, is a rare but potentially life-threatening necrotizing soft tissue infection (NSTI) of the genital, perineal, or perianal regions and demands immediate medical attention due to its aggressive nature.^1^ This disease has a reported overall mortality rate of 20–40 %, reaching 70–80 %, particularly if the patient presents with a septic profile.^2^ Therefore, it remains a challenging condition for healthcare providers globally^2^ FG predominantly affects adult males, with a peak incidence between the ages of 50 and 60 years.^3^ However, it can also occur in women and children, with a male to female ratio of 10:1.^4^

The precise aetiology of FG often involves a polymicrobial infection, with a mixture of aerobic and anaerobic bacteria contributing to its pathogenesis.^5^ The infection typically arises from a primary source, such as perineal trauma, urinary tract infections, or genital surgeries, facilitating bacterial infiltration into the subcutaneous tissues and subsequent necrosis. Furthermore, immunocompromised states, diabetes mellitus, obesity, and other comorbidities predispose individuals to this condition.^3^^,^^4^^,^^6^ Few cases of FG in adults after circumcision have been published.^7^, ^8^, ^9^, ^10^, ^11^

Understanding the definition, epidemiology, and causes of FG is paramount for early recognition and prompt initiation of aggressive treatment, which often involves surgical debridement and broad-spectrum antibiotic therapy. Failure to intervene swiftly can lead to rapid progression, systemic complications, and high mortality rates, underscoring the urgency of effective management strategies.

In this paper, we describe a case of FG that occurred in a 32-year-old male after voluntary circumcision due to phimosis. The patient had no previous medical history and no predisposing factors for the development of FG.

## 2 Case presentation

A 32-year-old male patient presented at the Emergency Department two days after circumcision in another clinic, with unbearable pain and swelling of the penis. Progressive pain and swelling of the penis developed one day after planned circumcision performed for phimosis. No signs of infection were observed at the time of the initial procedure. At the ED patient had a temperature of 38.1 °C, a heart rate of 126 per minute and a blood pressure of 102/68 mmHg. Inspection of the penis showed evident swelling and erythema with purple discoloration of the entire penis (Fig. 1A, Fig. 1BA and B). The scrotum, perineum and inguinal region showed no involvement. The patient had an elevated white blood cell count (12.0∗10ˆ9/l), C-reactive protein (160 mg/l) and sedimentation rate (25 mm/h). Initially the patient was treated with intravenous antibiotics, namely amoxicillin/clavulanic acid. However, due to increased pain, swelling, blistering, progression to the scrotum and a suspicion of a NSTI, the antibiotic treatment was directly altered. The patient received Meropenem and Clindamycin with an additional single dose of Tobramycin.

**Fig. 1A:**
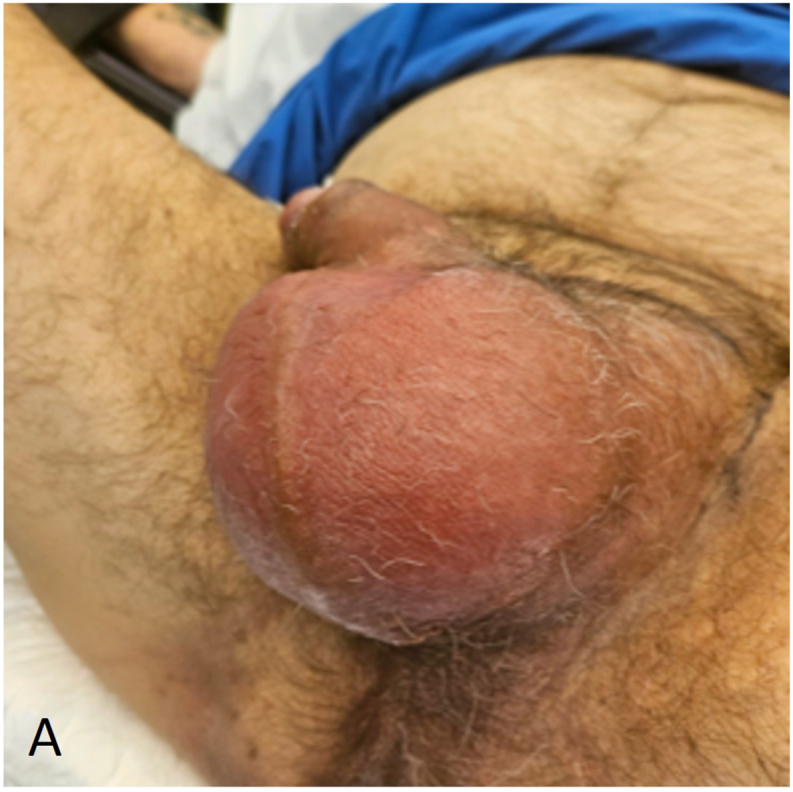
Clinical status at presentation to the emergency department two days after the initial circumcision, prior to surgical debridement.

**Fig. 1B:**
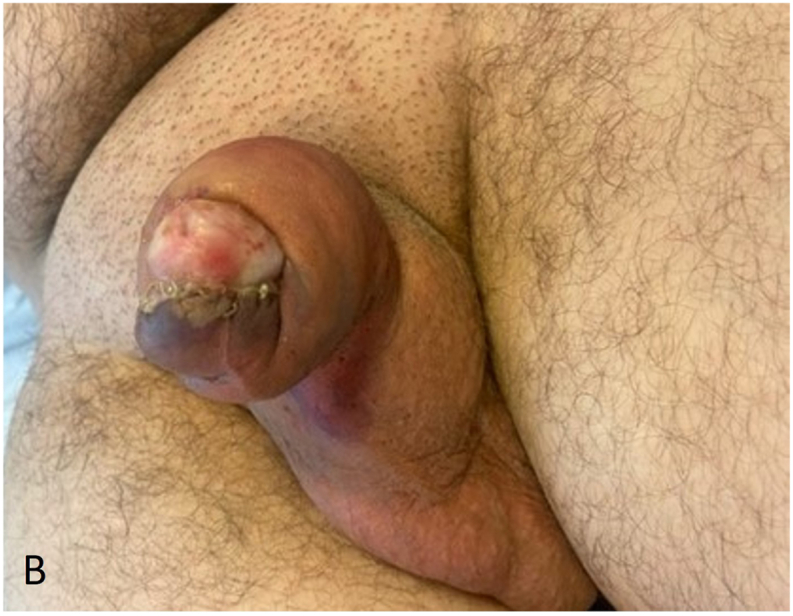
xxxxx

Five hours after initial presentation at the emergency department, the patient underwent surgical debridement in the operating room, during which the necrotic ventral skin of the penis and part of the scrotum were removed (Fig. 2A, Fig. 2BA and B). Fascia inspection through a suprapubic incision revealed no involvement. Following the procedure, the patient remained intubated and was admitted to the intensive care unit. Subsequently, the patient developed septic shock, requiring fluid resuscitation and high doses of vasopressors. In addition to antibiotics, intravenous immunoglobulin therapy were continued for three days. Due to hemodynamic instability and progressing necrosis, manifested by purple discoloration of the wound edges, repeated surgical debridement was performed in the operating room 8 h after the first surgical debridement. A negative pressure therapy device was placed for the prepubic wound (Fig. 3A, Fig. 3B).

**Fig. 2A:**
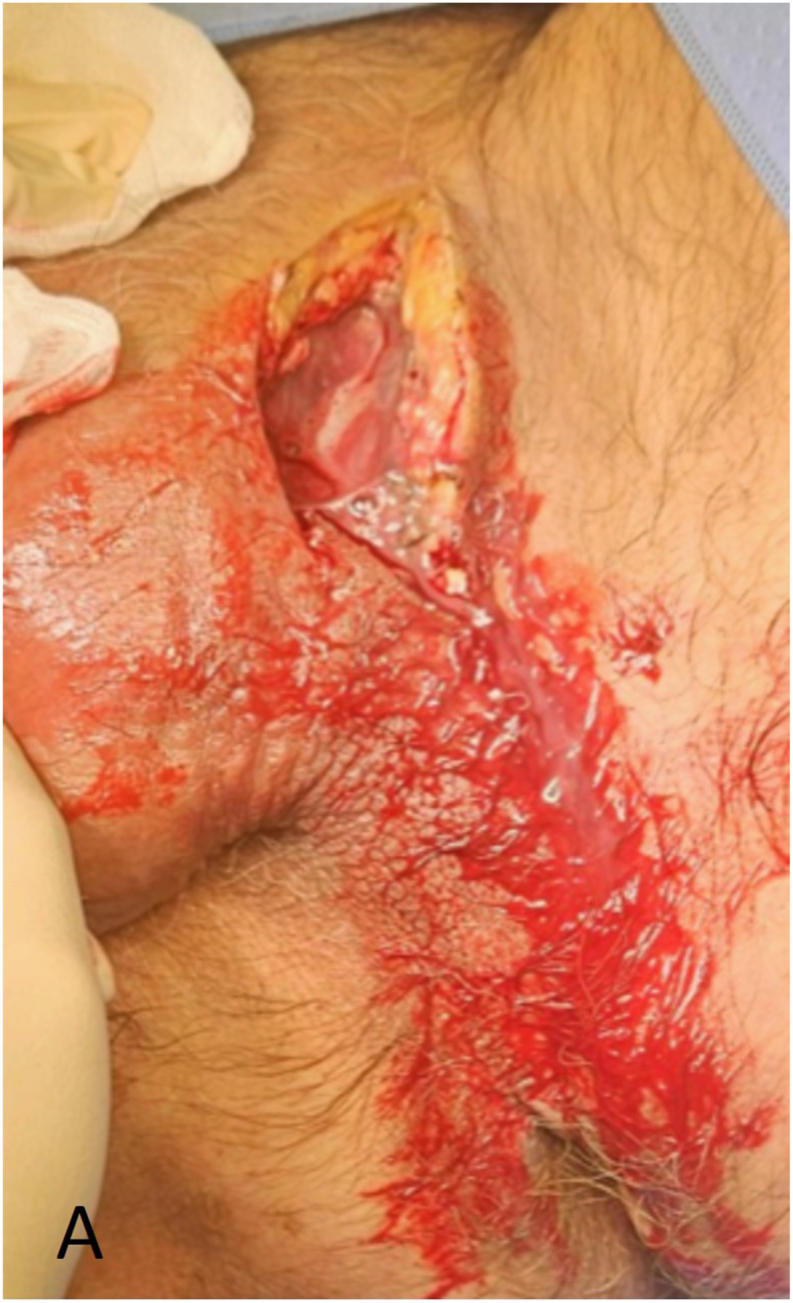
During the first operation, 5 h after initial presentation at the emergency department, the patient underwent surgical debridement in the operating room, necrotic ventral skin of the penis and part of the scrotum were removed.

**Fig. 2B:**
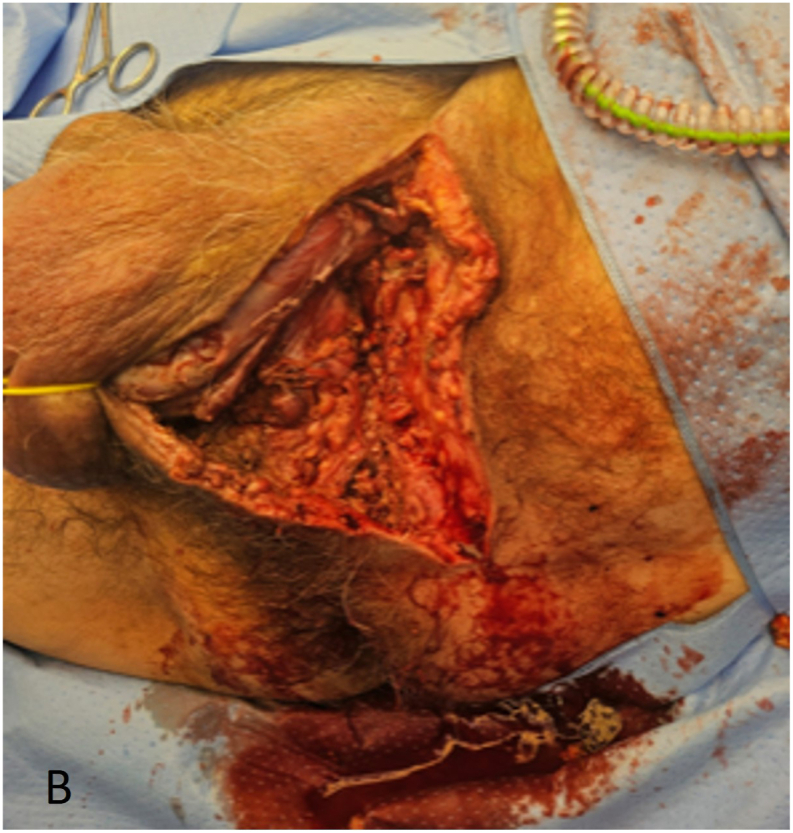
xxx

**Fig. 3A:**
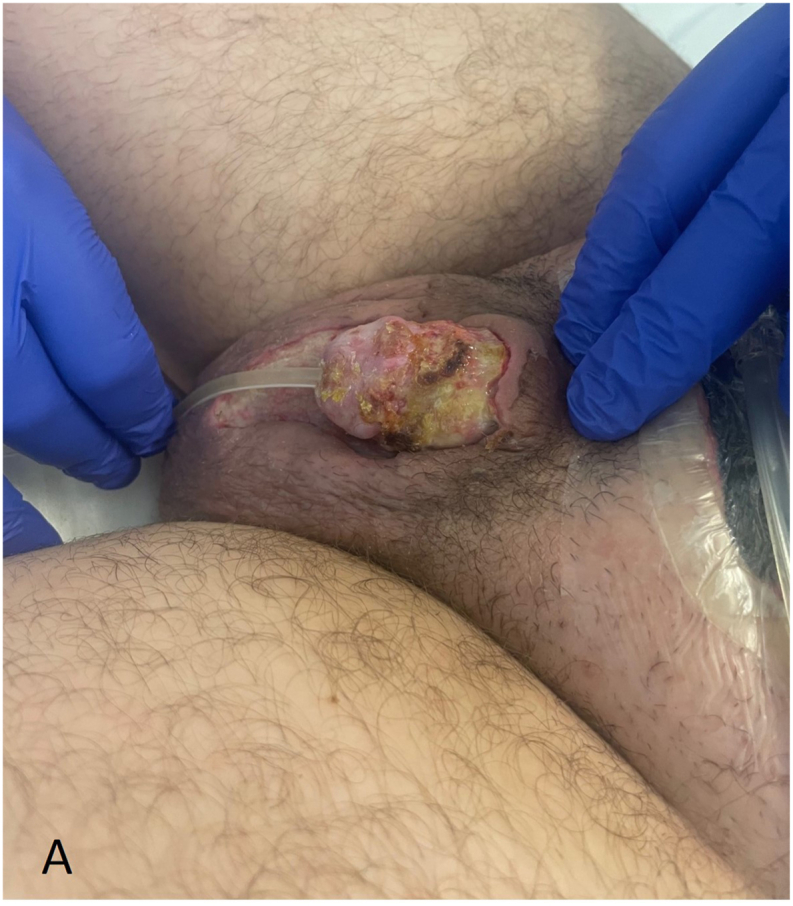
During early recovery: a negative pressure therapy device was placed on the prepubic wound after a second surgical debridement in the operating room (8 h after initial debridement).

**Fig. 3B:**
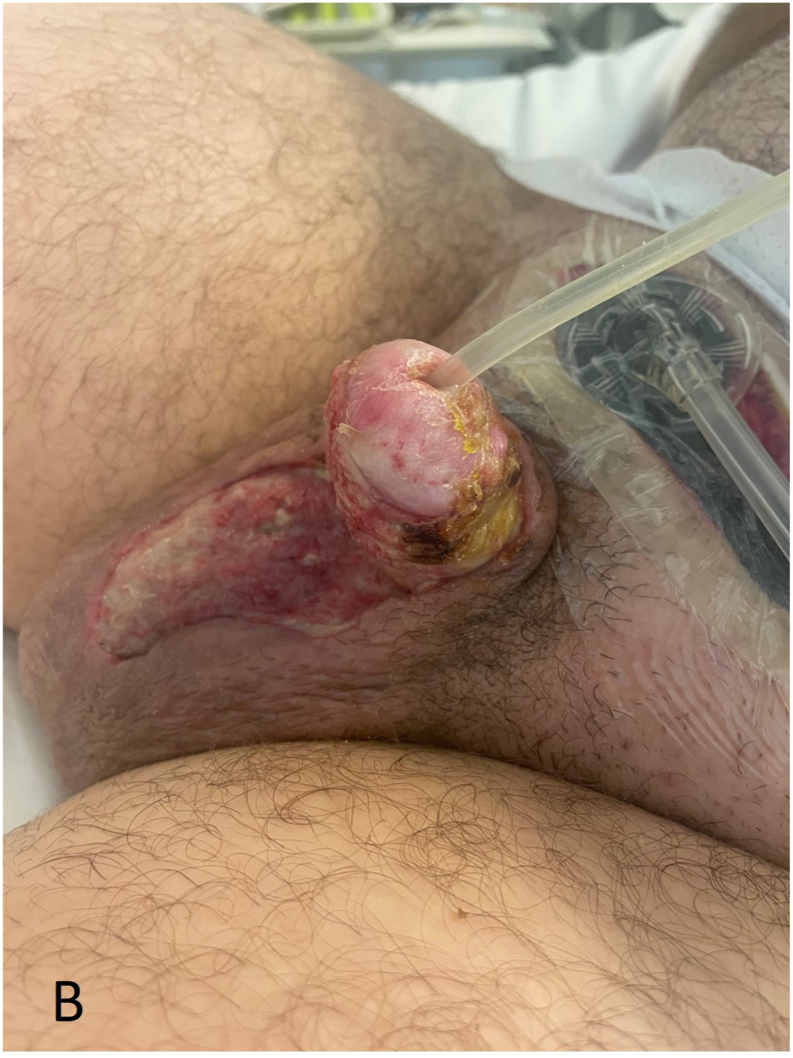
xxx

Perioperative tissue and wound cultures revealed the presence of *Staphylococcus aureus* and Group A Hemolytic Streptococci. The latter is known as a classical microbe present in NSTI, whereas *Staphylococcus aureus*, as cause of NSTI, is also frequently seen in post-operative wound infections. Consequently, the antibiotic regimen was modified to intravenous flucloxacillin for 10 days and clindamycin for 5 days. The patient's hemodynamic status improved, and the wounds showed signs of vitality. Two days later the patient was successfully extubated following a gradual reduction of sedation. The patient was discharged to the surgical ward in clinically stable condition for ongoing treatment and recovery.

The patient was transferred to a specialized (burn) center for specialized wound care where negative pressure therapy was reapplied to the prepubic wound. The suprapubic wound was primarily closed with sutures. Five days later, the wound bed was found to be vital enough for the placement of a split-thickness skin graft (SSG). The SSG was harvested from the patient's right upper leg using a 1:1 mesh technique. Excessive necrotic tissue surrounding the penis and scrotum was removed. Subsequently, the SSG was sutured onto the wound, and a negative pressure therapy device was applied to promote healing. Four days after adherence of the SSG, the device was removed and the patient was discharged from the hospital. In the subsequent weeks, the patient demonstrated significant recovery, with the suprapubic, penile, and scrotal wounds healing effectively (Fig. 4A, Fig. 4B). During recovery, the patient reported sequelae including painful erections, fatigue, and concentration disorders, which improved in the subsequent months.

**Fig. 4A:**
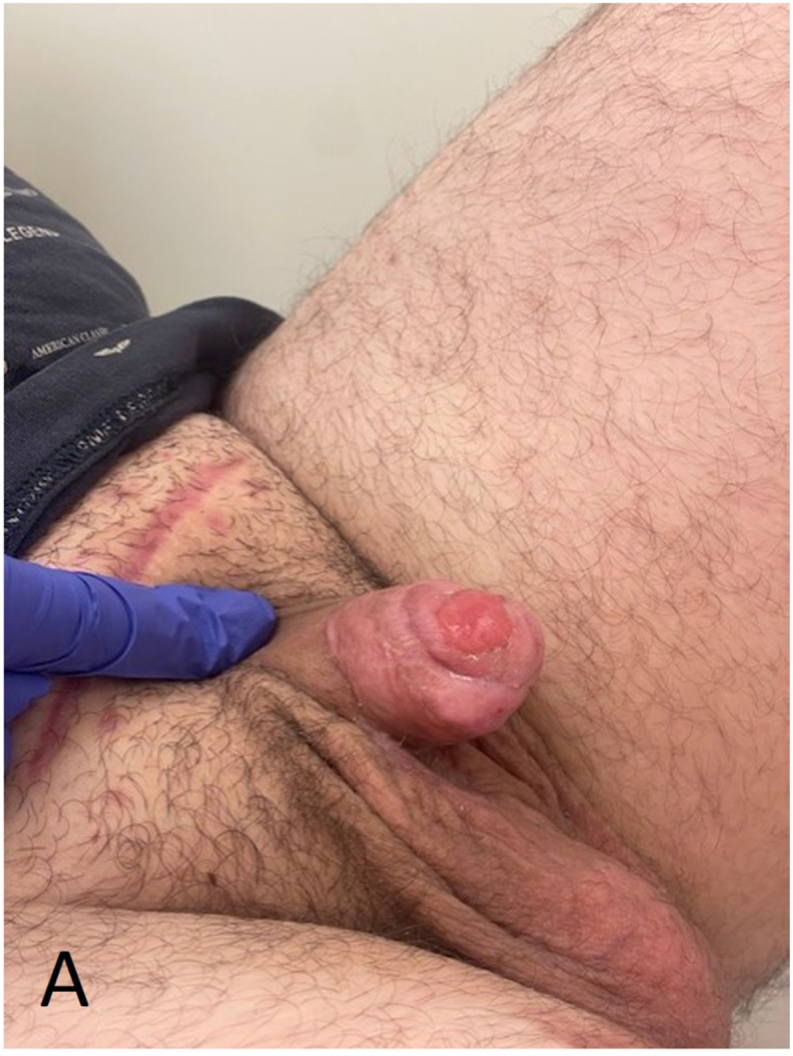
Late recovery of Fournier's gangrene after placement of a split-thickness skin graft.

**Fig. 4B:**
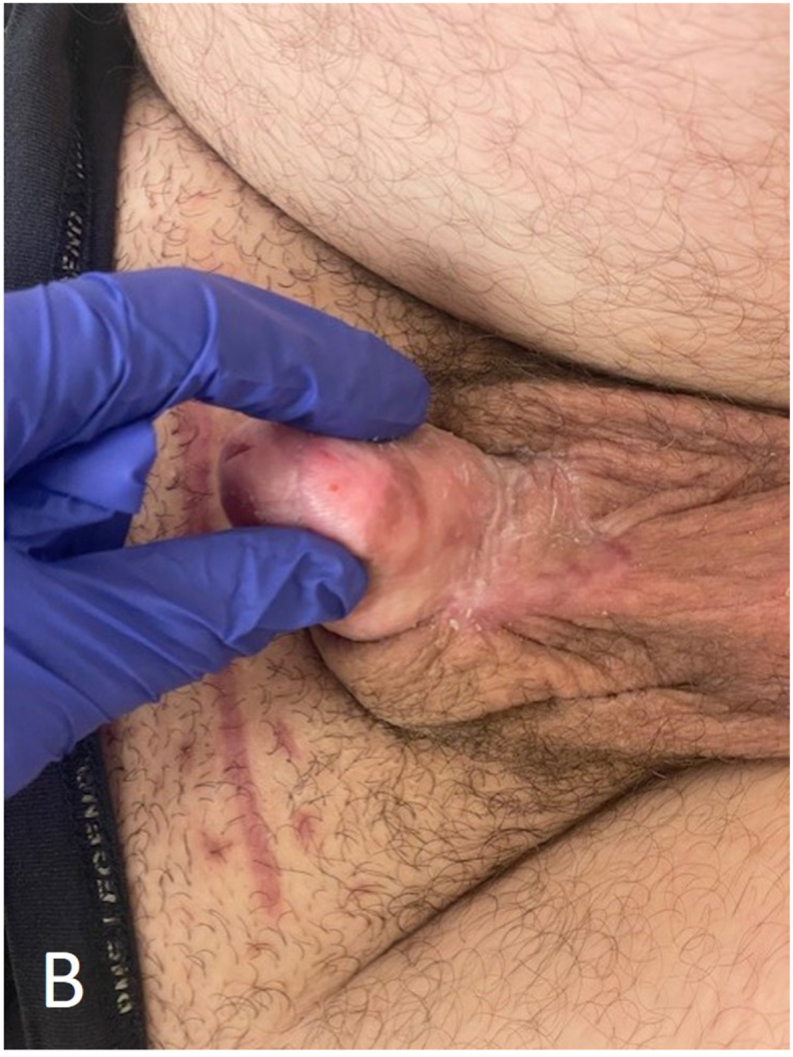
xxx

## 3 Discussion and conclusions

FG is a rare but life-threatening condition characterized by necrotizing infection of the genital, perineal, and perianal regions. The diagnosis is made on clinical grounds. While the exact aetiology of FG is multifactorial, several predisposing factors have been identified in the literature. These include diabetes mellitus, immunosuppression, obesity, alcoholism, and peripheral vascular disease.^3^^,^^4^^,^^6^ Additionally, prior circumcision has been proposed as a potential risk factor for the development of FG.^12^ However, the literature describing this relationship is limited with only a few case reports available.^7^, ^8^, ^9^, ^10^, ^11^ In the above-described patient, none of the aforementioned risk-factors were present and the circumcision was an elective procedure due to phimosis, conducted according to standardized care. Peri-operatively antibiotics were not given, according to current guidelines and sterile disinfection was conducted three times: initially preoperatively before administering local anesthetics, subsequently before the incision and after the incision. Soit seems that in this regard, no additional measures could have been taken to prevent this rare complication.

The association between prior circumcision and FG can be explained by several mechanisms. Circumcision, particularly if performed in a non-sterile environment or with inadequate postoperative care, can introduce pathogens into the surgical wound, leading to infection.^13^ Moreover, circumcision may result in compromised blood supply to the genital region, due to the vasoconstrictive local anaesthesia, arterial vasospasm, excessive use of monopolar electrosurgery or tight bandaging. The decreased blood supply predisposes individuals to tissue ischemia and subsequent infection.^14^^,^^15^ Once the patient develops FG, the infectious organisms initiate an inflammatory reaction leading to endarteritis. This blockage leads to the formation of thrombosis in the vessels that supply nutrients to the affected area, reducing blood flow and causing tissue ischemia. The decreased oxygen levels in the tissues encourage the growth of anaerobic bacteria, which in turn leads to necrosis and breakdown of the fascia.^16^^,^^17^ Although the development of FG following circumcision is rare and only described in a few case reports, clinicians should remain vigilant, in patients with and without predisposing factors.

Symptoms of FG typically include severe pain, erythema, edema, and crepitus in the affected area. Systemic signs such as fever, tachycardia, low blood pressure and elevated inflammatory markers may also be present as an expression of septic profile. Given the rapid progression of the disease, early diagnosis is crucial to prevent further tissue necrosis and systemic complications. Diagnosis of FG is primarily clinical, supported by radiological imaging such as computed tomography (CT) scans. CT imaging allows for the assessment of the extent of tissue involvement, identification of subcutaneous emphysema, and detection of underlying pathology.^4^^,^^12^ The presence of subcutaneous emphysema is not pathognomonic but is present in 90 % of patients with FG.^18^ A CT scan can be a useful diagnostic tool to expedite decision-making in unclear cases. In this case, it was not performed due to rapid clinical deterioration - marked by worsening erythema, severe pain, and blistering - which necessitated immediate surgical exploration. While a CT scan at initial presentation might have provided additional insights, it is unlikely to have significantly altered the treatment course given the urgency of the situation.

Microbiological analysis of tissue specimens obtained during surgical debridement often reveals a polymicrobial infection, with a mixture of aerobic and anaerobic bacteria. Commonly isolated micro-organisms include Streptococcus, *Staphylococcus aureus*, Enterococcus, *Escherichia coli*, and Bacteroides species.^5^ In this case, both Streptococci and Staphylococci were cultured, which is in line with previous literature. Surprisingly, no gram-negative rods or anaerobic bacteria were found, while a combination of these are frequently cultured in FG.^4^^,^^19^

Treatment of FG involves a multidisciplinary approach, including aggressive surgical debridement, broad-spectrum antibiotic therapy, and supportive care.^16^ Surgical debridement aims to remove necrotic tissue and control the spread of infection, often requiring multiple procedures to ensure complete eradication of the infection. A recent review by McDermott et al. emphasizes the importance of early surgical debridement, recommending intervention within 6 hours of presentation to optimize outcomes, even if the diagnosis is unconfirmed.^20^ This mirrors the aggressive treatment approach applied to our patient. Immediate broad-spectrum antibiotics, refined by tissue culture results, are critical in managing NSTIs, alongside hemodynamic support for septic shock when necessary.^12^ The aforementioned treatment options were all applied to our patient. Additional treatments such as hyperbaric oxygen therapy have been described to enhance tissue healing by improving fibroblast proliferation and neutrophil functioning.,^15^ however lack sufficient evidence for routine use. The prognosis of FG depends on several factors, such as extent of tissue involvement, underlying comorbidities, and timing of (surgical) intervention.

In conclusion, FG is a rare but potentially devastating condition, in specific NSTI of the penis after circumcision. Despite advances in diagnosis and treatment, FG remains associated with significant morbidity and mortality rates, emphasizing the importance of early recognition and prompt management, even in the absence of comorbidities. Furthermore, a polymicrobial culture with both aerobic and anaerobic bacteria is not always found. Although FG is described as a rare adverse event, this case illustrates the importance of awareness, early diagnosis and treatment, in patients with and without predisposing factors. Further research is needed to improve our understanding of FG and enhance treatment strategies for this condition.

## CRediT authorship contribution statement

**Isobel A. Bowring:** Writing – original draft, Conceptualization. **Lotte Bouwman:** Writing – review & editing, Writing – original draft, Investigation, Conceptualization. **Kim van Putten:** Writing – review & editing. **Joris J. Blok:** Writing – review & editing, Validation, Supervision, Conceptualization.

## Declarations

This study was partially conducted at the Leids University Medical Center.

## Ethics approval and consent for publication:written informed consent for publication was given by the patient

Ethics approval from the institutional review board was not applicable. Consent for publication and use of images for scientific purposes was given by the patient.

## Availability of data and materials

Not applicable.

## Funding

None.

## Declaration competing interests

The author's declare no competing interests.

## List of abbreviations

Fournier's gangrene (FG), necrotizing soft tissue infection (NSTI), computed tomography (CT), split-thickness skin graft (SSG).
